# Resistance to single-agent chemotherapy in low-risk gestational trophoblastic neoplasia

**DOI:** 10.22088/cjim.14.1.47

**Published:** 2023

**Authors:** Shahrzad Sheikhhasani, Aghdas Abdolrazaghnejad, Azam Sadat Mousavi, Setareh Akhavan, Narges Zamani, Elham Feizabad

**Affiliations:** 1 Department of Oncologic Gynecology, Vali-Asr Hospital, Tehran University of Medical Sciences, Tehran, Iran

**Keywords:** Single-agent chemotherapy, Gestational trophoblastic neoplasia, Dactinomycin, Methotrexate, Treatment failure

## Abstract

**Background:**

Methotrexate (MTX) and actinomycin D (ActD) have been used as first-line chemotherapy agents in the treatment of low-risk gestational trophoblastic neoplasia (GTN). Although low-risk GTN is considered a curable disease, its reported primary remission rates of 49 to 93% reflect the difficulties of treatment and different factors influencing it. Hence, this study aimed to determine the remission rates and related factors of single-agent chemotherapy resistance in low-risk GTN patients.

**Methods:**

This retrospective study included patients with diagnosed low-risk GTN who received either MTX once a week (IM, 30mg/m2) or ActD once every two weeks (pulsed IV, 1.25mg/m2). Then, the patients were followed-up until complete remission or single-agent treatment failure to assess resistance rate and related factors.

**Results:**

Eighty-four patients were included in the study (18 patients were receiving MTX and 66 patients were receiving ActD). 85.7% of all participants achieved complete remission after first-line chemotherapy (72.2% in MTX vs 89.4% in ActD). There was a significant association for higher tumor size (P=0.046), the occurrence of metastasis (P=0.019), and pretreatment β-HCG levels (P=0.005) with resistance to treatment.

**Conclusion:**

This study demonstrated higher tumor size, the occurrence of metastasis, and pretreatment β-HCG levels have been associated with increased resistance to first-line chemotherapy agents.

Gestational trophoblastic neoplasia (GTN) refers to malignant sequelae of pregnancies arising from the villous trophoblast, including choriocarcinoma, invasive mole, and placental site and epithelioid trophoblastic tumors. The prognosis for this type of malignancy is favorable with a close to 100% cure rate since the advent of an effective chemotherapy regime (1, 2). The incidence of gestational trophoblastic diseases which also include nonmalignant molar pregnancies varies on different geographical areas with higher rates observed in Indonesia (10/1000 pregnancies), and Mexico (4.6/1000 pregnancies) compared to North America and Europe (less than 1 in 1000 pregnancies (3). Certain races such as Hispanics are also less likely to develop GTN compared to the white population (4). The copious expression of beta-human chorionic gonadotropin (β-hCG) by the malignant cells allows for the use of its serum levels to diagnose, monitor, and follow- up responses to treatment. While GTN is diagnosed through rising β-HCG levels following pregnancies, the disease is further classified as either low or high-risk GTN based on the probability of drug resistance in the patients. 

Although several therapeutic regimens with chemotherapy agents have been developed, the optimal protocol for GTN treatment is still a matter of debate (1, 5). Both actinomycin D (ActD) and methotrexate (MTX) have been recommended for the initial course of adjuvant highly effective first-line single-drug chemotherapy in persistent low-risk GTN, though relevant guidelines have not reached a clear consensus on the preferential use of either of these drugs. Although both drugs are used in various doses and intervals (6-9), the most commonly used regimen is the weekly 30 mg/m2 body surface area for MTX and the biweekly 1.25 mg/m2 for ActD. Higher FIGO scores, choriocarcinoma diagnosis, and higher β-hCG levels have been associated with increased resistance to first-line treatments (10-12).

Although low-risk GTN is considered a curable disease, its reported primary remission rates of 49 to 93% reflect the difficulties of treatment and different factors influencing it (11). Hence, this study aimed to determine the remission rates and related factors of single-agent chemotherapy resistance in low-risk GTN patients. 

## Methods

This single-center retrospective cohort study was conducted on patients referred to or under treatment for low-risk GTN between 2011 and 2020 in Imam Khomeini Hospital, Tehran, Iran. This study was conducted in compliance with the Helsinki Declaration and approved by the Tehran University of Medical Sciences Ethics Committee. The participants signed the informed consent.

Patients were included in the study if they had been diagnosed with low-risk GTN by the International Federation of Gynecology and Obstetrics (FIGO) criteria: one or more of a < 10% decrease in three weekly β-hCG values (over 2 weeks); a > 20% rise in the β-hCG value over any two consecutive weekly assays; an elevated β-hCG level ≥ 4 months following initial uterine curettage; metastatic disease in the vagina, parametrium, or lung. 

The participants were deemed low-risk using FIGO cancer committee scoring system. A score of 0-6 was determined to be low risk in this study and suitable for single-agent chemotherapy with Act D or MTX. MTX or ACT-D were prescribed according to their gynecologist opinion, the drug availability, the patient preference, the drug cost, and as well as the underlying patient diseases. Participants were excluded if they were not receiving single-drug chemotherapy that had incomplete records or with unknown disease outcomes. Written informed consent was obtained from each patient. Patients were either receiving MTX once a week (30mg/m2, i.m.) or ActD biweekly (1.25mg/m2, i.v.) until reaching undetectable β-HCG levels or confirming resistance to the drug. Data extracted from the medical records of each patient included patient age, primary tumor size, history of molar pregnancy, β-HCG range, gravidity and parity, metastatic involvement and size, and a number of metastatic foci upon diagnosis. In addition, liver, renal function, and blood toxicity were evaluated. Outcomes included the association of tumor size and chemotherapy regimen with the initial response to a single course of chemotherapy. Resistance to treatment was defined as similar β-HCG levels (less than 10% variations) in two consecutive weekly assessments or increased β-HCG levels under treatment. Patients were followed-up with monthly β-HCG assays for a year after reaching undetectable serum levels to screen for disease relapse. The patient data were initially assessed for heterogeneity of variables in each chemotherapy group and subsequently for association with drug resistance using Pearson chi-square, or Fisher’s exact test when appropriate. 

The association between chemotherapy course and cure status was evaluated using a multivariate logistic regression model. Study variables were included as covariates if they rejected the null hypothesis at the 0.25 alpha level based on the Wald chi-square statistic in the univariate analysis and were significant in the multivariate with an alpha of 0.1 or resulted in a significant change in parameter estimates (13). The odds ratio and 95% confidence interval were then reported for the chemotherapeutic agents in the study after adjustment for selected covariates in the multivariate model. All analyses were performed in IBM Statistical Package for the Social Sciences (SPSS) Version 24.

## Results

Eighty-four patients were included in the analysis. 18 patients were following a regimen of MTX, while 66 patients were receiving an ActD treatment course. 63 (95.3%) and 15 (83.3%) of the patients were below the age of 40 in the ActD and MTX groups, respectively. A history of prior molar pregnancy was positive 37 (56.1%) and 11 (61.1%) women in ActD and MTX group, respectively. Metastasis of the primary tumor had occurred in 3 patients in the ActD group and 2 patients in the MTX group. All cases of metastasis were limited to the lung parenchyma. No cases of disease relapse with either of the regimen were documented. Baseline variables, β-HCG levels, the occurrence of metastasis, and tumor size was not statistically different between the groups (table 1). Toxicity was assessed in 84 patients, no deaths occurred related to toxicity. No pancytopenia was diagnosed in the patients, while nausea and vomiting, rising in AST, ALT, and alopecia were reported in 36, 3, 2, and 26 women, respectively; with no significant (p>0.05) differences between the two study groups except for alopecia which was significantly (P=0.009) higher in ActD treatment group.The analysis revealed significant association higher tumor size (P=0.046), occurrence of metastasis (P=0.019), and pretreatment β-HCG levels (P=0.005) with resistance to treatment regimen. Other variables including age (P=0.587), gravidity (P=0.914), live births (P=0.978), abortion (P=0.825), molar pregnancy (P=0.543), ectopic pregnancy (P=0.999) were not significantly associated with resistance to chemotherapy regimens. 

**Figure 1:**
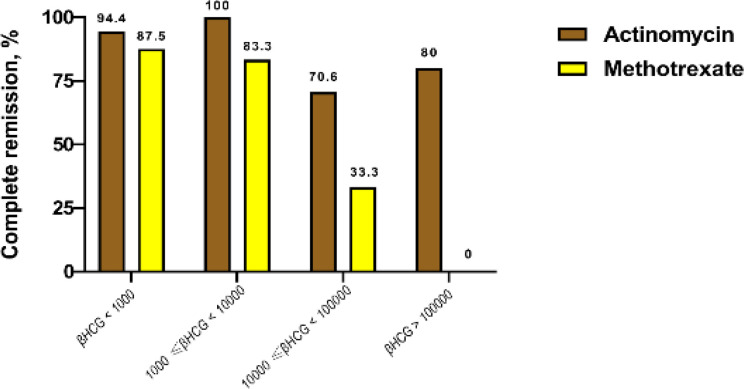
Cure rate across pretreatment HCG levels for actinomycin D and methotrexate

The results demonstrated significantly lower cure rates with MTX compared to ActD (72.2% vs 89.4%, respectively), corresponding to an odds ratio of 0.044 (95% confidence interval (CI): 0.003-0.586; P=0.018) after adjustment for age, primary tumor size, presence of metastatic foci, and β-HCG range. Figure 1 demonstrates the distribution of cases and cure rates among different serum levels of β-HCG. 

**Table 1:** Patient demographics, obstetrics history, and GTN characteristics

Variables	Total (n=84)	Chemotherapy agent		P-value
		Actinomycin D (N=66)	Methotrexate (N=18)	
**Age**				
**Age<40**	78 (92.9%)	63 (95.5%)	15 (83.8%)	0.109
**Age≥40**	6 (7.1%)	3 (4.5%)	3 (16.7%)	
**Gravidity**				
**1**	17 (20.2%)	14 (21.2%)	3 (16.7%)	0.586
**2**	20 (23.8%)	14 (21.2%)	6 (33.3%)	
**3**	29 (34.5%)	24 (36.4%)	5 (27.8%)	
**4**	14 (16.7%)	10 (15.2%)	4 (22.2%)	
**5**	4 (4.8%)	4 (6.1%)	0 (0.0%)	
**History of Live births**				
**0**	21 (25.0%)	17 (25.8%)	4(22.2%)	0.767
**1**	33 (39.3%)	26 (39.4%)	7 (38.9%)	
**2**	24 (28.6%)	5 (27.8)	5 (27.8%)	
**3**	4 (4.8%)	2 (11.1%)	4 (4.8%)	
**4**	1 (1.2%)	0 (0.0%)	1 (1.2%)	
**5**	1 (1.2%)	0 (0.0%)	1 (1.2%)	
**History of Abortion**				
**0**	39(46.4%)	30 (45.5%)	9 (60.0%)	0.414
**1**	35 (41.7%)	29 (43.9%)	6 (33.3%)	
**2**	9 (10.7%)	6 (9.1%)	3 (16.7%)	
**3**	1 (1.2%)	1 (1.5%)	0 (0.0%)	
**History of neonatal death**				
**0**	78 (92.9%)	60 (90.9%)	18 (100.0%)	0.688
**1**	5 (6.0%)	5 (7.6%)	0 (0.0%)	
**2**	84 (100.0%)	1 (1.5%)	18 (100.0%)	
**History of molar pregnancy**	48 (57.1%)	37 (56.1%)	11 (61.1%)	0.792
**Primary tumor size**				
**<3cm**	65 (77.4%)	51 (77.3%)	14 (77.8%)	0.283
**3-5cm**	15 (17.9%)	13 (19.7%)	2 (11.1%)	
**>5cm**	4 (4.8%)	2 (3.0%)	2 (11.1%)	
**Metastasized malignancy**	5 (6%)	3 (4.5%)	2 (11.1%)	0.290
**Pre-evacuation β-hCG levels (mIU/ml)**				
**<1000**	26 (31%)	18 (27.3%)	8 (44.4%)	0.561
**1000-10000**	32 (38.1%)	26 (39.4%)	6 (33.3%)	
**10000-100000**	20 (23.8%)	17 (25.8%)	3 (16.7%)	
**>100000**	6 (7.1%)	5 (7.6%)	1 (1.2%)	

* Difference in distribution of variables between groups was assessed using Fisher’s exact or Pearson chi-square test.

**Table 2:** Association of study variables with resistance to single-agent chemotherapy in low-risk GTN

Variables	Study population		P-value
	Complete remission(N=72)	Resistant to chemotherapy (N=12)	
**Age**			
**Age<40**	66 (91.5%)	12 (100%)	0.587
**Age≥40**	6 (8.3%)	0 (0%)	
**History of abortion**			
**0**	33 (45.8%)	6 (50%)	0.825
**1**	31 (43.1%)	4 (33.3%)	
**2**	7 (9.7%)	2 (16.7%)	
**3**	1 (1.4%)	0 (0.0%)	
**History of molar pregnancy**	40 (55.6%)	8 (66.7%)	0.543
**History of ectopic pregnancy**	1 (1.4%)	0 (0%)	0.999
**Primary tumor size**			
**<3cm**	59 (81.9%)	6 (50%)	0.046
**3-5cm**	10 (13.9%)	5 (41.7%)	
**>5cm**	3 (4.2%)	1 (8.3%)	
**Metastasized malignancy**	2 (2.8%)	3 (25%)	0.019
**Pre-treatment β-hCG levels (mIU/ml)**			
**<1000**	24 (33.3%)	2 (16.7%)	0.005
**1000-10000**	31 (43.1%)	1 (8.3%)	
**10000-100000**	13 (18.1%)	7 (58.3%)	
**>100000**	4 (5.6%)	2 (16.7%)	

* Fisher’s exact/Pearson chi-square test was performed on all of the study population to assess the significant association between resistance to treatment and study variables.

## Discussion

This study demonstrated complete remission and cure status in low-risk GTN was 89.4% in ActD compared to 72.2% in MTX. The results of this study are in line with the previous studies and systematic reviews in which biweekly ActD was associated with a higher complete remission rate compared to different regimens of MTX (3, 8, 14-17). Complete remission rates in patients receiving IV ActD in our study are in accordance with the results of previous studies, which reported ActD remission rates ranging between 70-94% (7, 8, 14, 18). The analysis also demonstrated a significant association for a number of prognostic factors, namely primary tumor size, presence of metastasis foci, and pretreatment β-HCG levels with treatment resistance. While FIGO scores have already been designed as a valuable multimarker factor for calculating the risk of failure with single-agent therapy, our results also suggest the importance of individual markers with the risk of resistance. Overall, metastatic disease has also been associated with resistance to single-agent chemotherapy in several studies and WHO classification (10, 11, 19). However, metastasis limited to lung tissue does not increase the classification score (19, 20). Despite this, resistance to treatment was higher in the limited number of patients with metastatic lung disease in our study compared to the overall study population (60% vs 14%, respectively) regardless of the administered chemotherapy agent. 

The association between lung metastasis in GTN with mortality, disease relapse, or additional chemotherapy has also been documented in previous studies (20), suggesting the need for additional research to evaluate the addition of lung metastasis as an independent prognostic factor for GTN. Despite promising results of the previous studies, MTX with or without folic acid rescue has historically been the preferred single-agent regimen for the primary therapy of low-risk GTN in several centers due to its proven effectiveness and ease of access (1, 15). ActD is therefore reserved as a second-line therapy for patients with resistance or contraindications to MTX use such as those with preexisting hepatic complications. Despite the reportedly increased incidence of side effects with ActD, such as alopecia, nausea, tissue injury, and hyperemesis, this agent was well-tolerated in the patients in this study. Moreover, no cases of cessation of treatment due to drug-related toxicities were observed for ActD in a considerable proportion of studies (16, 21, 22). 

Furthermore, the benefits of other MTX regimens with potentially higher efficacy compared to the weekly MTX assessed in this study were offset by increased treatment costs and outpatient’s visits in other studies comparing different regimens of these two agents (23). Despite the limitation of this study such as the small sample size, single-institutional and retrospective nature, our study demonstrated higher rates of achieving complete remission for ActD after adjusting for baseline and tumor characteristics of the participants. The ever-growing evidence of higher efficacy of ActD should be taken into consideration in patient-tailored clinical decision-making and treatment plans for low-risk GTN. Finally, this study demonstrated higher tumor size, the occurrence of metastasis, the number of previous neonatal death, and pretreatment β-HCG levels were associated with increased resistance to first-line treatments. 
